# Cerebral amyloid angiopathy, brain iron concentrations, and cognitive decline in older people

**DOI:** 10.1007/s00401-026-03056-9

**Published:** 2026-07-21

**Authors:** Sonal Agrawal, Maude Wagner, Sue E. Leurgans, Puja Agarwal, Louise van der Weerd, David A. Bennett, Ashley I. Bush, Scott Ayton, Julie A. Schneider

**Affiliations:** 1 Rush Alzheimer’s Disease Center, Rush University Medical Center, Jelke Building, 1750 W. Harrison Street, Chicago, IL 60612 USA; 2 Department of Pathology, Rush University Medical Center, Chicago, IL USA; 3 Department of Neurological Sciences, Rush University Medical Center, Chicago, IL USA; 4 Department of Internal Medicine, Rush University Medical Center, Chicago, IL USA; 5 Department of Radiology & Human Genetics, Leiden University Medical Center, Leiden, The Netherlands; 6 The Florey Institute of Neuroscience and Mental Health, The Florey Institute of Neuroscience and Mental Health, Parkville, VIC 3010 Australia

**Keywords:** Brain iron, Metal, Cerebral amyloid angiopathy, Small vessel disease, Alzheimer’s disease, Cognitive decline, Longitudinal cohort study

## Abstract

Cerebral amyloid angiopathy (CAA) is a common brain pathology in older people and has been recently recognized as a major risk factor for amyloid-related imaging abnormalities during anti-amyloid antibody therapy. CAA pathophysiology may involve iron released from ruptured vessels, but the association between postmortem CAA and brain iron is unclear. This study investigates the association between CAA and brain iron and whether elevated iron modifies the association between CAA and cognitive decline. We studied 626 Rush Memory and Aging Project decedents (mean age at death = 90 [SD = 6.1] years, 70% women) who completed baseline and longitudinal cognitive assessments and underwent detailed neuropathologic evaluation for CAA, Alzheimer’s disease neuropathologic changes (ADNC), and other brain pathologies. Brain iron content was assessed from the inferior temporal cortex using Inductively Coupled Plasma Mass Spectrometry (ICP-MS). Linear regression and mixed-effects models were used for analysis. CAA was common: 266 (42%) had mild, 153 (24%) had moderate, and 75 (12%) had severe CAA. In analyses adjusted for demographics, intermediate/high ADNC, and other pathologies, the presence and severity of CAA were associated with elevated cortical brain iron (Est = 0.033, SE = 0.011, *p* = 0.002; Est = 0.017, SE = 0.004, *p* < 0.001, respectively). When examining associations with nonlinear cognitive change before death (mean follow-up = 7.7 [SD = 3.9] years), both CAA and elevated iron were independently associated with faster annual rates of decline in global cognition and semantic memory (all *p* < 0.04). Elevated iron was also associated with faster declines in episodic and working memory and perceptual speed (all *p* < 0.02). When exploring whether brain iron modulates the association of CAA with cognitive decline, we found that CAA had steeper decline in perceptual speed when elevated iron was present compared to when low iron was present (*p* = 0.03). Together, these findings suggest that brain iron may contribute to the clinical impact of CAA in older age.

**Supplementary Information:**

The online version contains supplementary material available at 10.1007/s00401-026-03056-9.

## Introduction

Cerebral amyloid angiopathy (CAA) is a cerebrovascular disease characterized pathologically by amyloid-beta-peptide deposition in the media and adventitia layers of small- to medium-sized blood vessels in the brain and leptomeninges. CAA primarily affects the elderly, and the prevalence of CAA increases with age, affecting 20–40% of individuals over 60 years and as many as 80% of older decedents with pathologically confirmed Alzheimer’s disease (AD) [2, 9, 30]. CAA is also strongly linked to intracerebral hemorrhages, cerebral infarcts, and cognitive decline [8, 9, 19].

Brain iron accumulation has been recognized as a notable feature of aging and neurodegenerative processes and has been hypothesized to cause oxidative stress and cell death by ferroptosis [6, 52]. Quantitative techniques such as susceptibility‐weighted MRI and quantitative‐susceptibility mapping (QSM) have shown increased iron levels within the cortex of AD patients [25, 50]. Our prior postmortem study has also demonstrated that increased cortical iron is associated with cognitive decline in older individuals with pathologically confirmed AD [12]. Yet, lowering the brain iron with iron chelator causes marked accelerated cognitive decline in AD [14], revealing a prominent, albeit complex, role for iron in AD.

In contrast to AD, iron elevation in the brain may be contributed by CAA through bleeding of iron-rich blood products (especially hemoglobin) into the brain parenchyma [42] if so, iron elevation could report CAA pathology and its attendant links with cognitive decline. However, to date, it remains unclear whether brain iron dysregulation has any role in the effect of CAA on cognitive decline. Few studies have examined the association of CAA with brain iron [15, 42, 44]. Most of these studies have relied on MRI neuroimaging techniques to assess iron content and identify CAA in the brain, rather than direct examination of brain tissue. For instance, one study reported an association between CAA and elevated iron levels in cerebrospinal fluid but did not specifically examine iron within the brain [15]. Another study found no association between CAA and brain iron using quantitative susceptibility mapping [44]. In contrast, a histopathological study reported higher brain iron in individuals with severe CAA compared to controls, but this small study of 22 participants did not control for age, AD, or other potential confounders [42]. Given these studies with small numbers of participants and mixed results, further investigation is needed to understand the relationship between CAA and brain iron, and their effect on cognitive decline in aging. In this study, we extend prior research by testing the hypothesis that the presence and severity of postmortem CAA is associated with elevated cortical iron, and that the association between CAA and cognitive decline is influenced by cortical brain iron concentrations, independently of AD and other brain pathologies. To study these hypotheses, we utilized data from more than 600 autopsied participants from the Rush Memory and Aging Project (MAP), an ongoing clinical–pathologic epidemiological study focusing on aging and dementia.

## Methods

### Study participants

MAP is an ongoing longitudinal clinical–pathologic study of aging and dementia. MAP started in 1997 and enrolls older women and men living in retirement communities and housing facilities in the Chicagoland area [18]. Eligibility requires the absence of known dementia and agreement to annual clinical evaluations and to brain donation at death. To date, the follow-up rate exceeds 90%. Each participant provided informed consent, agreeing to annual cognitive evaluations and signing an Anatomic Gift Act form that authorized brain donation after death. The study received approval from the Institutional Review Board at Rush University Medical Center.

As of June 2025, 2471 MAP participants had enrolled and completed a baseline evaluation. Among these, 2095 individuals had undergone longitudinal cognitive assessments for at least two visits, 1279 had passed away, and 1111 had undergone brain autopsy, resulting in an autopsy rate of 87%. Of the 1111 participants who had brain autopsy, 1080 had complete neuropathologic evaluation with complete pathology data on CAA. Of these, 626 had postmortem cortical brain iron data available and were included in this study. Note that participants with dementias other than Alzheimer’s disease were not included in this study.

### Cognitive function assessment

Each annual clinical evaluation included a uniform medical history, neurologic examination, and detailed neuropsychological testing performed by examiners blinded to the prior clinical data. The cognitive battery consisted of 21 neuropsychological tests, of which 19 were used to create a composite measure of global cognitive function and specific summary measures for 5 cognitive domains: episodic memory, working memory, semantic memory, perceptual speed, and perceptual orientation. Individual test raw scores were converted to *z*-scores based on the mean and standard deviation derived from the baseline evaluations of the overall cohort, allowing for the calculation of summary scores for each cognitive domain. Subsequently, these standardized *z*-scores were averaged across the 19 tests to yield the composite score representing global cognitive function [26, 53]. A lower composite score indicates poorer cognitive performance.

### Neuropathologic assessment

At autopsy, the brain was removed, weighed, and cut into 2 hemispheres. The average postmortem interval of brain autopsy was 6.7 (interquartile range 5.6–8.6) h. One hemisphere was cut coronally immediately into 1 cm slabs and reviewed for any suspected infarcts. The slab with suspected pathology was fixed in 4% paraformaldehyde for neuropathologic evaluation and the rest of the slabs were frozen in a − 80 °C freezer for biochemical studies, including cortical brain iron. The other hemisphere was fixed in 4% paraformaldehyde for at least 1 month. After a month, the hemisphere was coronally cut into 1-cm slabs and reviewed for atherosclerosis and suspected gross infarcts. The evaluations were conducted by examiners blinded to all clinical data, allowing for an unbiased examination. The standard brain regions were dissected into blocks, embedded in paraffin, and cut into 6-μm sections for brain neuropathologies assessment [40].

#### Cortical brain iron

As previously reported [13], brain iron concentrations were measured in the frozen tissue (≈ 50 mg) from the inferior temporal cortex of 626 postmortem brains. Tissue samples were cut into 50 ± 5 mg using a ceramic blade to avoid metal contamination. These specimens were weighed, then homogenized using Tris-buffered saline containing phosphatase and protease inhibitors. The tissue samples were stored at − 80 °C until analysis. Iron levels from the brain tissues were measured using ICP-MS (Ultramass 700, Varian). Tissue samples from each experiment were freeze-dried and then resuspended in 69% nitric acid (ultraclean grade, Aristar) overnight. The samples were then heated for 20 min at 90 °C, and equivalent volume of hydrogen peroxide (30%, Merck) was added for a further 15 min incubation at 70 °C. The samples were diluted in double-distilled water and assayed by ICP-MS. Each tissue sample was measured in triplicate and the concentrations determined from the standard curve were normalized to wet tissue weight. The concentration of brain iron is expressed as µg/g. In this study, the brain iron distribution exhibited skewed distributions, and we applied the logarithm base 10 transformation for all analytic analyses.

#### Cerebral amyloid angiopathy

As described previously [19], CAA was assessed in both parenchymal and meningeal vessels using immunostained tissue from four brain regions: midfrontal, middle temporal, inferior parietal, and calcarine cortices, employing a monoclonal 4G8 antibody (1:9000; Bio legend, San Diego, CA). Each region was assigned a score ranging from 0 to 4, reflecting the degree of Aβ deposition present in the vessels. The regional amyloid scores were determined by taking the maximum scores from the parenchymal and meningeal CAA scores and averaging these across the four neocortical regions to create a mean score categorized as none, mild, moderate, and severe, based on the cutoffs (none when average is 0, mild when average < 1.5, moderate when average is 1.5–2.5, and severe when average is > 2.5) determined by the neuropathologist. For both descriptive and analytic analyses, a binary classification of presence and absence of CAA as well as a four-level CAA severity classification (none, mild, moderate, and severe) were used.

#### Alzheimer’s disease

For the pathologic diagnosis of AD, the National Institute on Aging-Alzheimer’s Association criteria relied on Braak, CERAD, and Thal stages were used [29]. Bielschowsky silver stain was used to visualize neuritic plaques and neurofibrillary tangles in five regions: midfrontal, middle temporal, inferior parietal, and entorhinal cortices, and hippocampus to evaluate CERAD neuritic plaque severity scores and Braak NFT stage as described previously [41]. In addition, Thal phase was determined from seven areas of the brain: midfrontal, middle temporal, and inferior parietal cortices, hippocampus CA1, basal ganglia, midbrain, and cerebellum immunostained with monoclonal 4G8 antibody (1:9000; Bio legend, San Diego, CA). In the descriptive and sensitivity analyses, AD neuropathologic change (ADNC) was categorized into four levels: none, low, intermediate, and high ADNC. For the main analytical analyses, those with intermediate or high ADNC were grouped and classified as having pathologically confirmed AD, whereas those with no or low ADNC were grouped as not having pathologically confirmed AD [4]. This approach has been widely used in previous studies for defining the neuropathological diagnosis of AD [1, 16, 31].

#### Other age-related neuropathologies

Meguro staining for iron detection in the inferior temporal cortex [49] and other neurodegenerative pathologies included amyloid-beta load, tau-tangle density, the presence of limbic-predominant age-related TDP-43 encephalopathy–neuropathologic change (LATE-NC; stages 2 and 3) and neocortical Lewy bodies; vascular pathologies included the presence of moderate/severe arteriolosclerosis, moderate/severe atherosclerosis; chronic macro- and micro-infarcts were evaluated using standardized methods as described in the supplementary method [1, 5, 7, 32, 34–37, 41].

### Assessment of other covariates

Other covariates included age at death, sex, years of formal education, and the presence of APOE-Ɛ4 genotype (at least one ℇ4 allele) determined by the Broad Institute for Population Genetics using the Polymorphic DNA Technologies [54]. In addition, we considered the number of vascular disease risk factors (diabetes, hypertension, smoking history) and the number of vascular disease conditions (heart conditions, stroke, congestive heart failure, claudication) self-reported proximate to death, as described previously [10, 37, 43].

### Statistical analyses

Spearman correlation was used to examine bivariate associations of cortical brain iron with age and education. Analysis of variance (ANOVA) with Tukey’s studentized range tests and *t* tests were used to compare the cortical brain iron with sex, APOE-Ɛ4, ADNC, and CAA severity. Similarly, ANOVA with Tukey’s studentized range tests or chi-square tests were used to compare the severity of CAA with continuous (age and education) and categorial (sex, APOE-Ɛ4, and ADNC) terms.

First, we employed linear regression models to test the hypothesis that the presence of postmortem CAA is associated with higher levels of cortical brain iron. All models were adjusted for age at death, sex, and education. Next, we extended the model by including terms for ADNC and other brain pathologies (neocortical Lewy bodies, LATE-NC, arteriolosclerosis, atherosclerosis, macro- and micro-infarcts). Finally, the model was separately adjusted for APOE-ε4, number of vascular disease risk factors, or number of vascular disease conditions. In secondary analyses, we examined whether the association of CAA with cortical brain iron differed by modifiers of interest: sex, APOE-ε4 risk allele, vascular disease risk factors, and vascular disease conditions. We fitted a separate fully adjusted model for each modifier, as specified above, further including an interaction between CAA and the modifier.

Second, we employed two separate linear mixed models to test two hypotheses: (i) CAA severity and higher cortical brain iron levels are independently associated with rates of cognitive decline before death; and (ii) brain iron levels modify the associations between CAA and cognitive change before death. For hypothesis (i), the model was adjusted for brain iron levels (continuous) and CAA severity (continuous), both as simple effects and in interaction with the time function. In this study that examines a wide range of neuropathologies, we have chosen to model CAA severity with only one degree of freedom in the longitudinal analyses; whether we treated CAA severity as a categorical or continuous variable did not affect the observed associations (results not shown). For hypothesis (ii), we further included the Iron by CAA interaction term, both as simple effects and in interaction with the time function. To capture possible accelerated changes in cognition over time, cognitive trajectories were modeled using natural cubic splines with two inner knots located at the tertiles of measurement times; the nonlinear trajectories approximated by splines provided substantially better fits than linear trajectories (e.g., Akaike information criteria = 3858.8 [splines] and = 4921.9 [linear], respectively, when testing hypothesis [i]). The models were adjusted for age at death, sex, education, and the presence of ADNC and other neuropathologies, both as simple effect and in interaction with splines functions of time; a time varying term for mode of cognitive administration (generally in person, however, during COVID pandemic, via telephone) was also included; interindividual variability was captured by correlated random intercept and slope. In secondary analyses, we tested the same hypotheses for each of the five cognitive domains. The basis of splines is defined through a combination of parameters, which are difficult to interpret individually. Thus, we have plotted the marginal estimated cognitive trajectories for an average study participant profile and provided the *p* value from the multivariate Wald test for the predictors of interest (CAA and iron) in interaction with the spline parameters. Finally, we performed a path analysis to assess the extent to which the associations of CAA severity and with decline in global cognition before death can be attributable to brain iron levels. The model partitioned the total association of CAA severity, with cognitive decline into a direct association and an indirect association through brain iron levels, which were estimated by standardized path coefficients.

Analyses were conducted using SAS/STAT software version 9.4 (SAS Institute Inc, Cary, NC) and R software version 4.0.3 (R Foundation for Statistical Computing). For linear mixed models, we used the hlme function of lcmm R package version 1.7.8 [38]. For mediation analysis, we used the PROC CAUSALMED [48]. Statistical significance was set at *p* < 0.05, two-sided.

## Results

### Participants

The analytic sample included 626 MAP decedents, predominantly women (70%), with a mean of 15 (SD = 2.8) years of education and a mean age at death of 90 (SD = 6.1) years (Table 1). At autopsy, the mean concentration of cortical brain iron was 51.3 (SD = 13.9; IQR = [42.4–57.9]; range = 23.0;141.8) μg/g wet weight, and CAA was common (mild: 266 [42%]; moderate: 153 [24%]; severe: 75 [12%]). During a mean follow-up of 7.7 years (SD = 3.9) before death, a total of 4444 cognitive measures were assessed, with a mean of 7.1 (SD = 3.6) cognitive assessments per individual.

**Table 1:** Demographics, clinical and neuropathologic characteristics of participants at death, Rush Memory and Aging Project

Characteristics	All participants (*N* = 626)	CAA severity			
		None (*N* = 132)	Mild (*N* = 266)	Moderate (*N* = 153)	Severe (*N* = 75)
Age at death, years, mean (SD)	90.48 (6.09)	89.69 (6.40)	90.59 (5.80)	90.51 (6.33)	91.44 (6.01)
Women, No. (%)	441 (70.45)	93 (70.45)	193 (72.56)	101 (66.01)	54 (72)
Education, years, mean (SD)	14.59 (2.82)	14.96 (2.69)	14.49 (2.74)	14.57 (2.93)	14.33 (3.12)
APOE-Ɛ4 carriers, No. (%)	159 (25.44)	13 (9.85)	54 (20.38)	51 (33.33)	41 (54.67)
Number of vascular risk factors^a,b^, mean (SD)	1.28 (0.79)	1.35 (0.83)	1.31 (0.80)	0.77 (0.90)	1.11 (0.69)
Number of vascular disease conditions^a,c^, mean (SD)	0.86 (0.95)	0.81 (0.87)	0.92 (0.98)	1.24 (0.78)	0.91 (1.08)
Cognitive status^a^, No. (%)					
No cognitive impairment	199 (31.79)	60 (45.45)	84 (31.58)	43 (28.10)	12 (16)
Mild cognitive impairment	159 (25.40)	34 (25.76)	80 (30.08)	33 (21.57)	12 (16)
Alzheimer’s dementia	268 (42.81)	38 (28.79)	102 (38.35)	77 (50.33)	51 (68)
Cognitive function^a^, mean (SD)					
Global cognition	− 0.97 (1.13)	− 0.54 (0.96)	− 0.92 (1.10)	− 1.13 (1.15)	− 1.55 (1.16)
Episodic memory	− 0.90 (1.27)	− 0.43 (1.16)	− 0.83 (1.26)	− 1.11 (1.26)	− 1.54 (1.23)
Semantic memory	− 0.82 (1.30)	− 0.47 (1.06)	− 0.74 (1.17)	− 1.02 (1.47)	− 1.36 (1.53)
Working memory	− 0.78 (1.13)	− 0.46 (1.02)	− 0.75 (1.11)	− 0.88 (1.17)	− 1.22 (1.16)
Perceptual speed	− 1.12 (1.01)	− 0.84 1.06)	− 1.09 (0.99)	− 1.24 (0.89)	− 1.49 (0.99)
Perceptual orientation	− 0.61 (1.09)	− 0.39 (1.04)	− 0.61 (1.04)	− 0.65 (1.19)	− 0.95 (1.10)
Clinical interval^d^, years, median [IQR]	0.78 (0.44–1.33)	0.96 (1.14)	1.31 (1.55)	1.36 (1.61)	1.18 (1.26)
Brain iron level, inferior temporal cortex, μg/g, mean (SD)	51.25 (13.87)	48.32 (11.79)	50.93 (13.77)	52.25 (15.63)	55.53 (12.75)
ADNC (intermediate/high), No. (%)	418 (66.77)	45 (34.09)	182 (68.42)	128 (83.66)	63 (84)
LATE-NC, No. (%)	217 (34.89)	38 (29.01)	87 (32.83)	59 (39.07)	33 (44)
Neocortical Lewy bodies, No. (%)	79 (12.62)	17 (12.88)	33 (12.41)	22 (14.38)	7 (9.33)
Atherosclerosis, No. (%)	192 (30.67)	42 (32.06)	90 (33.83)	49 (32.03)	31 (41.33)
Arteriolosclerosis, No. (%)	212 (33.92)	43 (32.58)	84 (31.58)	42 (27.45)	23 (30.67)
Macroscopic infarcts, No. (%)	243 (38.82)	55 (41.67)	102 (38.35)	54 (35.29)	32 (42.67)
Microscopic infarcts, No. (%)	189 (30.19)	40 (30.30)	78 (29.32)	41 (26.80)	30 (40)

*APOE* apolipoprotein E, *ADNC* Alzheimer’s disease neuropathologic change, *CAA* cerebral amyloid angiopathy, *LATE-NC* limbic-predominant age-related TDP-43 encephalopathy–neuropathologic change, *SD* standard deviation

^a^Assessed proximate to death

^b^Diabetes, hypertension, and smoking history

^c^Heart conditions, stroke, congestive heart failure, and claudication

^d^Years from last clinical evaluation to death

We previously reported that iron in the inferior temporal cortex was elevated in decedents who had clinical AD in this cohort and associated with cognitive decline [12]. In the current study, we confirm in bivariate analyses that elevated inferior temporal iron was negatively associated with global cognition (rho = − 0.168, *p* < 0.001). In addition, we observed brain iron was elevated in APOE-Ɛ4 carriers (*t* value = − 2.7, DF = 242.1, *p* = 0.007), in persons with pathologically confirmed AD (*t* value = − 2.0, DF = 495.3, *p* = 0.042), in those with LATE-NC (*t* value = − 1.8, DF = 620, *p* = 0.046), and in those with moderate to severe CAA (*t* value = − 3.0, DF = 624, *p* = 0.002). Cortical brain iron levels were not associated with neocortical Lewy bodies, macro- or microscopic infarcts, arteriolosclerosis, and atherosclerosis (all *p* > 0.07). As expected, CAA was associated with lower global cognition (*p* < 0.001) and positively associated with APOE-Ɛ4 (*χ*^2^ = 59.29, DF = 3, *p* < 0.001), ADNC (*χ*^2^ = 93.57, *DF* = 3, *p* < 0.001), but not with other brain pathologies (all *p* > 0.1).

### CAA and cortical brain iron level

In linear regression models, adjusted for age at death, sex, education, the presence of CAA in the brain was associated with elevated (log_10_-transformed) cortical brain iron (estimate = 0.033 μg/g, SE = 0.011, *p* = 0.002; Table 2). The associations remained significant after controlling for ADNC and other brain pathologies (Table 2). Within the same model, LATE-NC, Lewy bodies, and other cerebrovascular pathologies were not significantly associated with cortical brain iron, although the association with LATE-NC approached toward statistical significance (estimate = 0.018 μg/g, SE = 0.009, *p* = 0.051; Supplementary Table 1). Consistent with our prior study [12], a pathologic diagnosis of AD was also not associated with brain iron (*p* = 0.139); however, when ADNC was replaced by separate measures of amyloid and tau-tangles, higher tau-tangle density was associated with higher cortical brain iron (estimate = 0.023 μg/g, SE = 0.005, *p* < 0.001; Supplementary Table 1). Furthermore, when examining the association of each level of CAA severity with cortical brain iron, compared to decedents without CAA, we found that those with moderate CAA (estimate = 0.027 μg/g, SE = 0.013, *p* = 0.042; Table 2) and those with severe CAA (estimate = 0.060 μg/g, SE = 0.016, *p* < 0.001; Table 2) had elevated iron, after controlling demographics and other brain pathologies. However, decedents with mild CAA had no higher cortical brain iron levels (estimate = 0.020 μg/g, SE = 0.011, *p* = 0.090; Table 2) in comparison with those with no CAA. Histological sections showing CAA severity and iron burden from age-matched AD female participants are presented in Supplementary Fig. 1.

**Table 2:** Multivariable-adjusted associations between cerebral amyloid angiopathy (CAA) and the log10-transformed cortical brain iron levels, Rush Memory and Aging Project (*N* = 626)

Predictor	Outcome: cortical brain iron				
	Model A	Model B	Model C	Model D	Model E
Presence of CAA	0.033 (0.011, 0.002)	0.027 (0.011, 0.016)	0.025 (0.011, 0.027)	0.027 (0.011, 0.018)	0.031 (0.012, 0.007)
Adjusted *R*^2^, %	2.2	2.5	2.7	2.5	2.7
AIC	− 2167.47	− 2147.80	− 2143.66	− 2125.79	− 2033.19
CAA severity stage					
None	Ref.	Ref.	Ref.	Ref.	Ref.
Mild	0.023 (0.011, 0.041)*	0.020 (0.011, 0.090)	0.019 (0.011, 0.107)	0.019 (0.012, 0.097)	0.025 (0.012, 0.038)*
Moderate	0.033 (0.012, < 0.008)	0.027 (0.013, 0.042)*	0.026 (0.013, 0.051)	0.027 (0.013, 0.043)*	0.030 (0.014, 0.030)*
Severe	0.066 (0.015, < 0.001)	0.060 (0.016, < 0.001)	0.057 (0.016, < 0.001)	0.060 (0.016, < 0.001)	0.062 (0.016, < 0.001)
Adjusted *R*^2^, %	3.3	3.5	3.5	3.4	3.3
AIC	− 2173.15	− 2152.20	− 2146.71	− 2130.08	− 2034.10
CAA severity	0.019 (0.004, < 0.001)	0.017 (0.004, < 0.001)	0.016 (0.005, 0.001)	0.017 (0.005, < 0.001)	0.017 (0.005, 0.001)
Adjusted *R*^2^, %	3.5	3.7	3.6	3.6	3.5
AIC	− 2176.04	− 2154.92	− 2149.58	− 2132.90	− 2038.41

Values in cells represent estimated coefficients (SE, *p *value)

Model A: Adjusted for age at death, sex, and education

Model B: Model A + ADNC and other common neuropathologies (neocortical Lewy bodies, LATE-NC, arteriolosclerosis, atherosclerosis, macro- and micro-infarcts)

Model C: Model B + APOE-Ɛ4 allele

Model D: Model B + number of vascular disease risk factors

Model E: Model B + number of vascular disease conditions

^*^p values no longer significant after false discovery rate (FDR) correction

We next explored whether the associations between CAA and cortical brain iron were robust when accounting for other potential confounders. First, APOE-Ɛ4 was reported to be related to CAA [55]. Thus, we added a term for APOE-Ɛ4; the association did not change (Table 2). Second, since vascular risk factors/diseases may affect the association of CAA with cortical brain iron, we repeated our model by including terms for vascular risk burden and vascular disease burden; the association remained similar (Table 2). Finally, when examining whether the associations between CAA and cortical brain iron levels were moderated by APOE-Ɛ4 genotype, sex, vascular risk factors, and vascular conditions, we found no evidence of effect modification (data not shown, all *p* > 0.09).

### CAA, cortical brain iron level, and cognitive decline

**Fig. 1:**
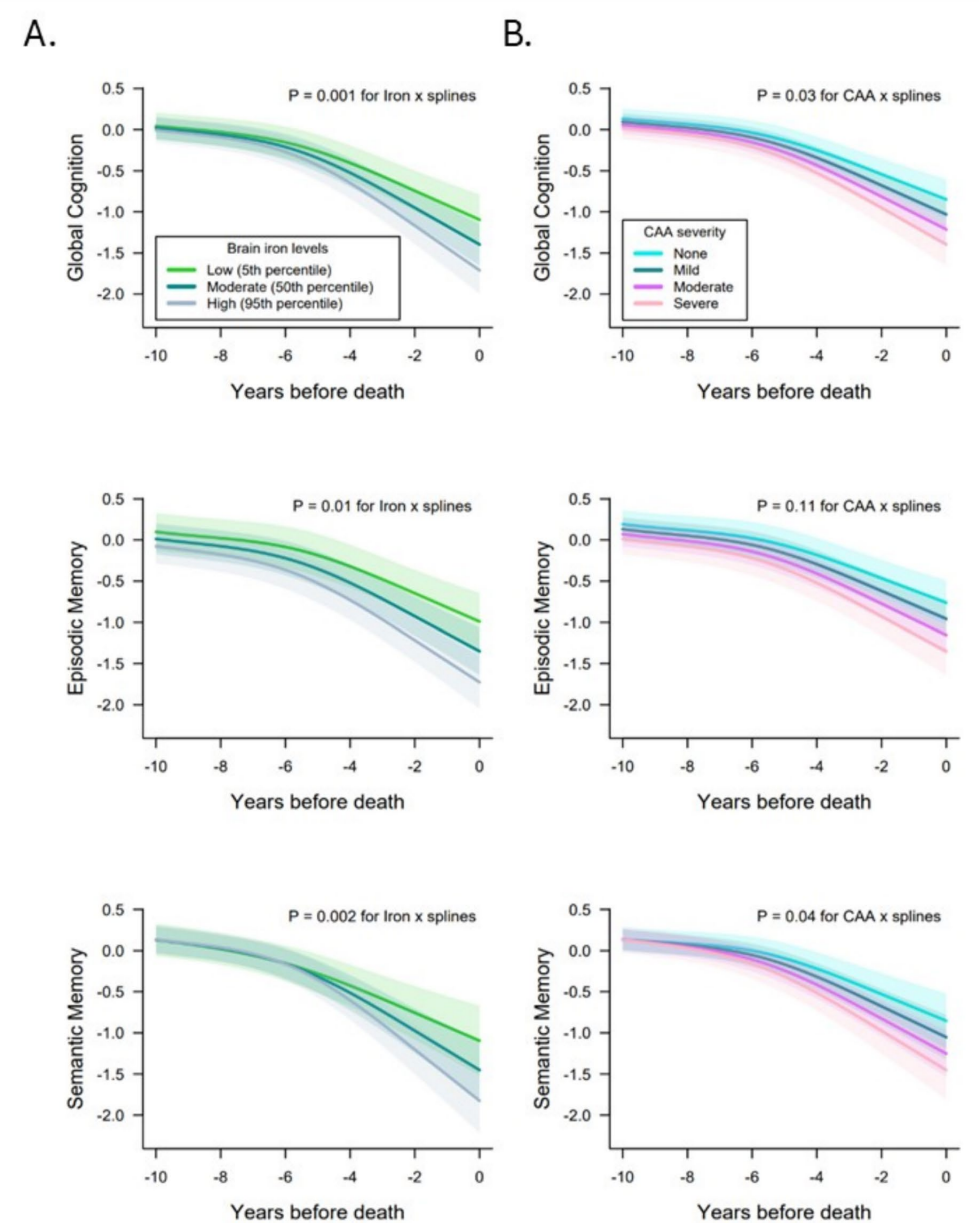

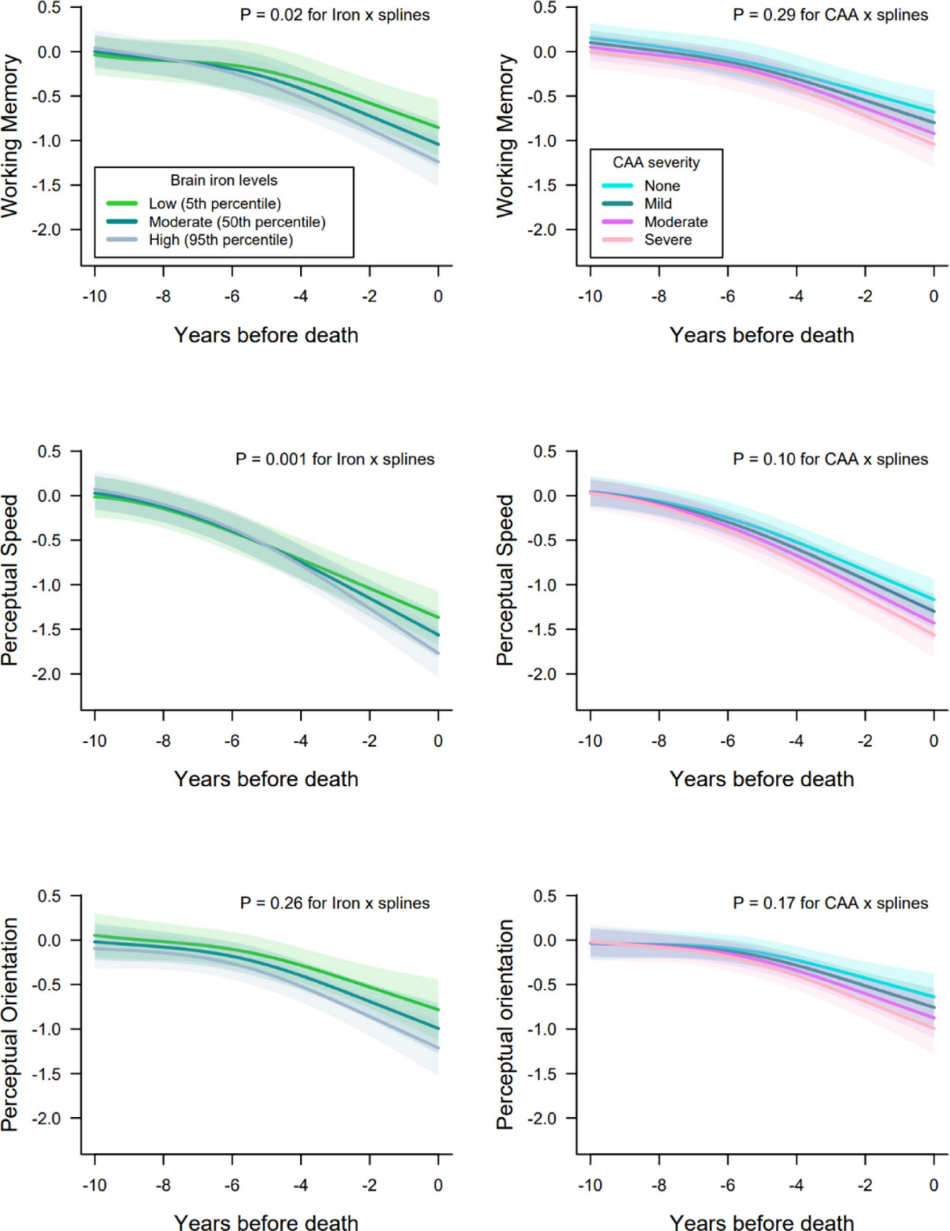
Mean estimated trajectories of change in global cognition and five cognitive domains in the years before death, by increasing levels of cortical brain iron (**A**, left panels) and CAA severity (**B**, right panels). Estimated trajectories are derived from six separate linear mixed models, with time before death approximated using natural cubic splines. Each model included: log_10_-transformed brain iron levels; CAA severity; covariates (demographics, mode of cognitive assessment, ADNC, LATE-NC, neocortical LBs, arteriolosclerosis, atherosclerosis, and cerebral infarcts); and the interactions of all variables with the splines time function. Curves represent the mean estimated trajectories (solid lines) and 95% confidence intervals (indicated with shadings) of an average study participant profile (a woman, aged 90 years at death, with 15 years of education, who had intermediate/high ADNC and chronic infarcts but no other pathologies). *p* values for CAA were no longer significant after FDR correction

Higher brain iron levels and increasing CAA severity were independently associated with faster annual rates of decline in global cognition before death (Fig. 1A: *p* = 0.001 for Iron × splines time function; Fig. 1B: *p* = 0.03 for CAA × splines time function). For example, the estimated accelerated change in global cognition for a 1-unit (μg/g) increase in brain iron levels was − 0.11 (95% CI − 0.15; − 0.08) standard units − 15 years before death, − 0.24 (95% CI − 0.26; − 0.22) standard units − 7 years before death, and − 1.78 (95% CI − 1.82; − 1.75) standard units at death (year 0). Similarly, for worse CAA severity, the estimated accelerated change in global cognition at − 15 years, − 7 years, and death were − 0.04 (95% CI − 0.03; − 0.05), − 0.05 (95% CI − 0.05; − 0.04), − 0.18 (95% CI − 0.19; − 0.17) standard units, respectively. When investigating the same associations for the cognitive domains, we found that higher brain iron levels were independently associated with faster decline in episodic memory, semantic memory, working memory, and perceptual speed (*p* = 0.01, = 0.002, = 0.02, = 0.001 for Iron × splines time function, respectively; Fig. 1A). We also found that worse CAA severity was independently associated with faster decline in semantic memory (*p* = 0.04 for CAA × splines time function; Fig. 1B). In sensitivity analyses, replacing the dichotomous ADNC classification with a four-level scheme (none; *N* = 94, low; *N* = 126, intermediate; *N* = 259, high; *N* = 147), the trend for CAA-related cognitive change remained generally unchanged. However, the associations between CAA and cognitive change (both global and semantic) were no longer statistically significant (Supplementary Fig. 2). These findings should be interpreted cautiously, as the attenuation may reflect reduced statistical power due to smaller ADNC subgroup sizes. Nonetheless, this finding suggests the importance of future studies that can disentangle the independent and combined contributions of CAA and ADNC to cognitive decline.

**Fig. 2:**
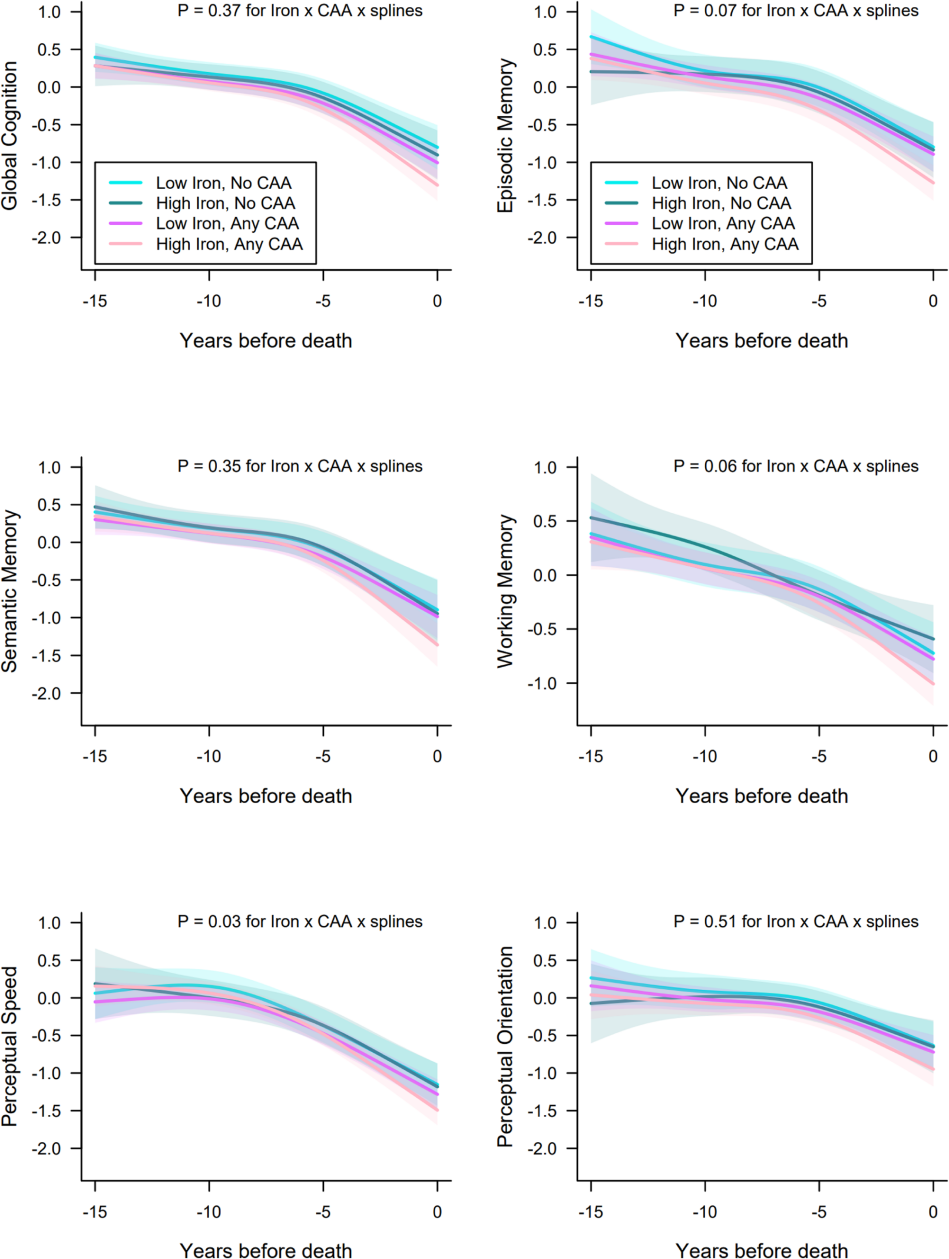
Mean estimated trajectories of change in global cognition and five cognitive domains in the years before death, by interaction between levels of cortical brain iron and the presence of CAA. Estimated trajectories are derived from six linear mixed models, with time before death approximated using natural cubic splines. Each model included: log_10_-transformed brain iron levels; presence of CAA; Iron by CAA interaction; covariates (demographics, mode of cognitive assessment, ADNC, LATE-NC, neocortical LBs, arteriolosclerosis, atherosclerosis, and cerebral infarcts); and the interactions of all variables with the splines time function. Curves represent the mean estimated trajectories (solid lines) and 95% confidence intervals (indicated with shadings) of an average study participant profile (a woman, aged 90 years at death, with 15 years of education, who had intermediate/high ADNC and chronic infarcts but no other pathologies). *p* values were no longer significant after FDR correction

Testing our second hypothesis, we did not find evidence that brain iron levels modify the association between the presence of CAA (Fig. 2) or CAA severity (Supplementary Fig. 2) and change in global cognition (*p* = 0.37 for Iron × presence of CAA × spline time function, Fig. 2; *p* = 0.57 for Iron × CAA severity × spline time function, Supplementary Fig. 3).

For the cognitive domains, we observed that brain iron levels modified the association between the presence of CAA and perceptual speed decline (*p* = 0.03 for Iron × presence of CAA × spline time function, Fig. 2), suggesting that those with higher brain iron levels, presence of CAA had steeper decline in perceptual speed than those with low iron levels. However, we did not observe any significant interactions of CAA with iron when we replaced the presence of CAA with the severity of CAA (all *p* > 0.09 for Iron × CAA severity × spline time function, Supplementary Fig. 3). The lack of interaction between iron and severity of CAA could be due to the small number of participants present with severe level of CAA in this study. Furthermore, we did not find evidence that brain iron levels modify the association between the presence of CAA (Fig. 2) or CAA severity (Supplementary Fig. 3) and other 4 cognitive domains (all *p* ≥ 0.07 for Iron × presence of CAA × spline time function, Fig. 2; all *p* ≥ 0.29 for Iron × CAA severity × spline time function, Supplementary Fig. 3).

### Exploratory analyses

We hypothesized that iron accumulation is an important factor in the cognitive impact of CAA. We performed path analyses to map the contribution of CAA to cognitive decline, focusing on the extent to which iron elevation accounts for these relationships as iron levels are associated with CAA and cognitive decline. We used person-specific slopes of change in cognition as an outcome and CAA as predictor and iron level as mediators and we hypothesized the 2 postulated steps—(1) a direct association of CAA with cognitive decline without going through iron, (2) an indirect association of CAA with cognitive decline through iron.

**Fig. 3:**
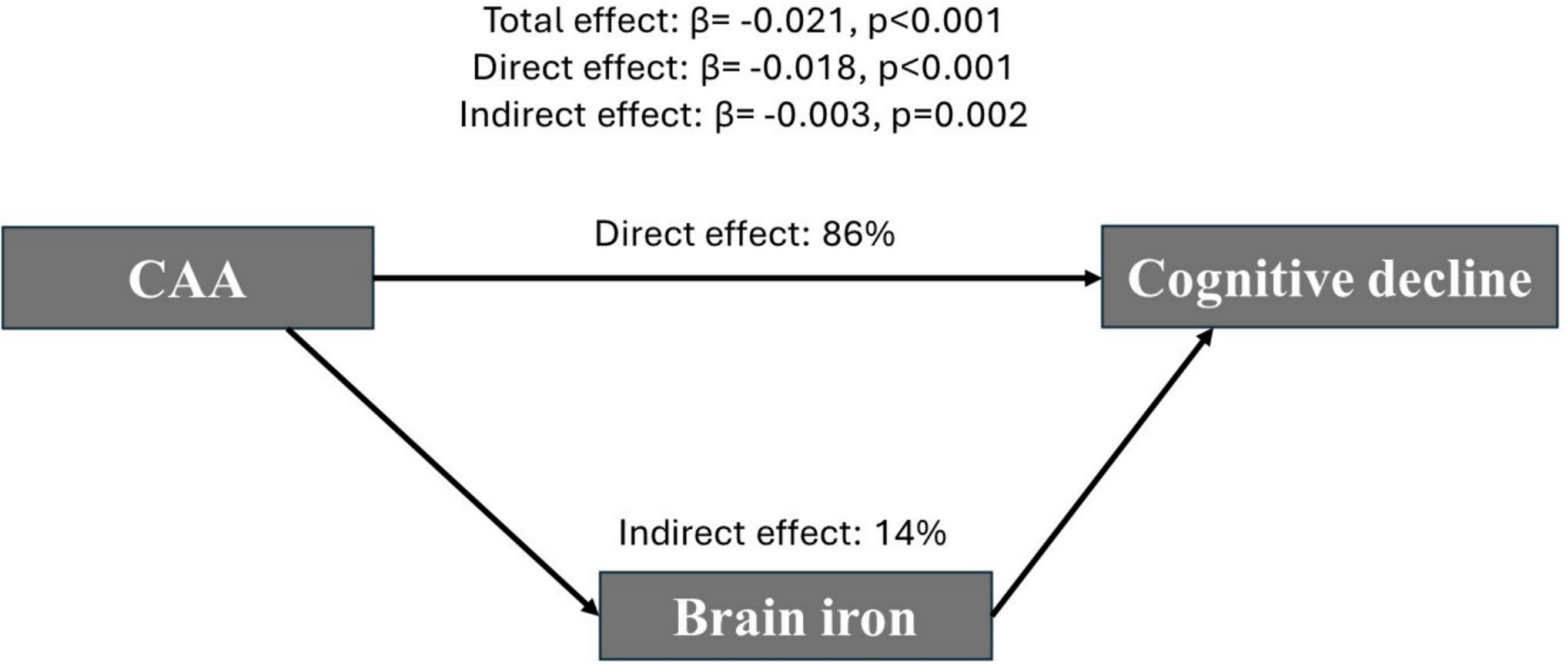
Pathway analysis mapping CAA severity, iron, and cognitive decline. The iron accumulation is a mediating variable between CAA and cognitive decline, after controlling for age at death, sex, and education, suggesting that 14% of the effect of CAA on cognitive decline can be explained by iron deposition in the brain

We found CAA was associated with cognitive decline (estimate = − 0.021, SE = 0.003; *p* < 0.001), with an indirect association of 14% through iron (estimate = − 0.003, SE = 0.001, *p* = 0.002, Fig. 3).

## Discussion

In this longitudinal study of 626 community-based older adults with a brain autopsy, we observed that both the presence and severity of CAA were associated with increased cortical iron levels, independent of AD and six other common dementia-related neuropathologies. CAA severity and elevated brain iron were each independently linked to more rapid cognitive decline after accounting for intermediate/high ADNC and other pathologies. Moreover, although more severe CAA was associated with accelerated cognitive decline across all levels of iron, among individuals with any CAA, those with elevated cortical iron exhibited a steeper decline in perceptual speed than those with low iron. This pattern indicates that brain iron impacts the clinical burden of CAA. We also found that less than 15% of the effect of CAA on cognitive decline was attributed to iron. Together, these findings demonstrate that CAA and brain iron are related yet independently contribute to cognitive decline. Brain iron both modestly mediates the impact of CAA and interacts with CAA to exacerbate cognitive deterioration, supporting the idea that CAA and iron share intersecting but distinct pathways leading to cognitive decline.

The role of brain iron elevation in AD pathogenesis has been widely appreciated through both animal and human postmortem studies [3, 23, 45–47]. Several postmortem studies have shown strong association between iron elevation and both pathological burden and spatial distribution of tau pathology over the cortex [20, 22] as well as contributions to cognitive decline particularly in those with pathologically confirmed AD [12]. Despite predictions, depleting brain iron with an iron chelator actually accelerated AD progression [14]. This unexpected outcome challenges the straightforward notion that high iron is solely detrimental and led to a hypothesis that elevated brain iron in AD might represent a functional iron deficiency, where iron is sequestered, e.g., within protein aggregates, rather than being bioavailable. In the present study, we specifically examined the relationship between CAA and brain iron in older people with and without pathologically confirmed AD and dementia. We found that increased CAA severity is associated with elevated cortical iron, even after accounting for pathologic diagnosis of AD and other brain pathologies, indicating that CAA itself may contribute to iron accumulation beyond the effects of AD and other neurodegenerative and cerebrovascular pathologies. Consistent with our findings, a prior study also reported higher nonheme iron in severe CAA cases [42], whereas another study found no association between CAA and iron and proposed that iron accumulation primarily contributed to AD [44]. This latter study, however, was using clinical diagnosis of CAA and compared with AD patients and normal controls (all which may have some level of CAA) and was not able to measure CAA and AD neuropathologies. These discrepant results may also be due to differences in the spatial distribution of iron. A study on hereditary cerebral hemorrhage with amyloidosis–Dutch type showed the evidence of iron deposits in the vessel wall [21]. Further investigation is needed to understand the spatial distribution of iron accumulation in CAA which may clarify the relationship of iron to AD compared to CAA pathology.

This study provides new data on large community-based older participants that extends the results of the previous studies by three significant ways [12, 19]. First, we demonstrate that both iron accumulation and CAA exert substantial and independent effects on cognitive decline. The potential independent mechanisms linking iron to cognitive decline may include ferroptosis, an iron-dependent regulated cell death pathway, mitochondrial dysfunction, and neuroinflammation [17, 28, 39]. Second, although the effect of CAA on cognitive decline is largely direct and independent of iron, we also identify a discernible indirect effect mediated by elevated iron levels, suggesting that iron dysregulation may represent an important biological process influencing cognitive function in individuals with CAA. Finally, at the domain-specific level, we observe a significant interaction between CAA and iron that is associated with greater decline in perceptual speed, indicating that iron accumulation may modify the cognitive profile of CAA. The mechanisms underlying this interaction remain unclear and require further study. However, one possible explanation could be that CAA and iron act through distinct pathological pathways, and their co-occurrence may lead to greater tissue or cellular damage, resulting in worse perceptual speed in older adults. Alternatively, iron-related processes such as ferroptosis and inflammation may increase tissue vulnerability for the toxic effects of CAA, thereby amplifying decline in perceptual speed.

Given that cross-sectional nature of the data in this study, it is difficult to determine whether CAA pathology precedes iron accumulation, or vice versa. However, three possibilities should be considered which suggest that CAA may contribute to iron accumulation. First, blood–brain barrier (BBB) disruption associated with CAA may permit leakage of fluid and neurotoxic plasma proteins into surrounding tissue, further impairs vascular reactivity, pericyte function, and oligodendrocyte proliferation, and thereby promoting iron accumulation [51]. Second, CAA may promote intravascular and perivascular inflammatory responses which may contribute to iron accumulation [11, 33]. Third, CAA-related dysfunction of the glymphatic clearance system may impair iron removal from the brain, ultimately leading to iron accumulation [24]. In addition, more studies are needed to determine whether CAA has any specific iron‐associated neuroinflammatory or neurovascular signatures that could improve antemortem diagnosis and inform the development of targeted therapies for CAA [27].

This study has multiple strengths. First, MAP assesses cognitive change over time by following participants longitudinally using a standardized cognitive test battery. Second, this study included a systematic neuropathologic assessment approach blinded to clinical data to examine the neurodegenerative and cerebrovascular measures which provide a definitive neuropathologic confirmed diagnosis. Finally, this is the largest study to examine the association of neuropathologically confirmed CAA with brain iron. This study also has limitations. First, MAP is restricted to older adults recruited by one team. MAP participants are predominantly non-Hispanic Whites with high education; all agreed to annual clinical evaluations and brain donation; thus, it will be valuable to replicate these findings in additional cohorts, especially from diverse populations. Second, the current study provides evidence of CAA association with brain iron and cognitive decline, but causal inferences are not possible. Third, although our statistical analyses were controlled for a wide range of potential confounders, unmeasured or residual confounding may be possible. Fourth, we included only one region for iron measurement (inferior temporal cortex); therefore, more focused analyses examining the relationship between iron accumulation and CAA particularly in regions, such as occipital cortex, which is strongly affected by CAA, may provide additional insight into the role of iron in CAA. Finally, it is plausible that elevated iron levels in individuals with CAA may, at least in part, reflect underlying hemorrhagic pathology. Unfortunately, details regarding chronic macroscopic and microscopic hemorrhages were not systematically collected throughout the course of the study, limiting our ability to evaluate this possibility. Future studies are warranted to determine the extent to which elevated brain iron in individuals with CAA is attributable to CAA-related microbleeds. In addition, perivascular hemosiderin, a well-known occurrence in brains both with and without focal intraparenchymal hemorrhage, may be a source of iron. Hemorrhagic and perivascular pathways should be considered as one of the possible pathways in the pathogenesis of elevated brain iron in future studies.

To conclude, the current study provides compelling evidence of a complex and important association between CAA and brain iron. While CAA and brain iron largely have separate association with cognitive decline, we found that a small component of CAA’s effect on cognitive decline appears to be attributed by iron. Finally, we also found synergy for CAA and iron specifically in decline in perceptual speed. These results advance our understanding of the role of iron dysregulation in CAA pathophysiology, and prompt new avenues for research.

## Supplementary Information

Below is the link to the electronic supplementary material.

Supplementary file1 (DOCX 1039 KB)

## Data Availability

Research data can be requested at Rush Alzheimer’s Disease Center Research Resource Sharing Hub (http://www.radc.rush.edu) to access all MAP data.
