# Supplementary material for: Cerebral amyloid angiopathy, brain iron concentrations, and cognitive decline in older people
Source: Acta Neuropathol. 2026 Jul 21;152(1):8. doi: 10.1007/s00401-026-03056-9 (PMC13388426; doi:10.1007/s00401-026-03056-9)

**SUPPLEMENTARY INFORMATION**

**Supplementary method:**

**β-amyloid load and PHF tau-tangles density:** Quantitative measures for β-Amyloid load and tau tangle density are based on historical and/or digital data from 8 brain regions including middle and superior frontal, inferior temporal, cingulate gyrus, inferior parietal, calcarine, and entorhinal cortices, and hippocampus. Cortical and hippocampal CA1-subiculum regions of interest were manually outlined. Digital image analysis for Aß pathology was quantified using the Aperio Image Analysis Toolbox Positive Pixel Count algorithm and digital image analysis for tau tangle pathology was quantified using the Aperio Image Analysis Toolbox Nuclear algorithm. Digital measures for bother measures are highly correlated with historical data and have been harmonized, as previously described [32].

**Limbic predominant age-related TDP-43 encephalopathy-neuropathologic change (LATE-NC):** LATE-NC was determined based on the presence of TDP-43 cytoplasmic inclusions using immunohistochemistry with a monoclonal antibody against phosphorylated TDP-43 (pS409/410; BioLegend, San Diego, CA; dilution 1:10,000). Eight brain regions including amygdala, hippocampal dentate gyrus and CA1–subiculum, entorhinal, middle temporal, anterior temporal pole, midfrontal, and orbitofrontal cortices were examined [34]. LATE-NC staging was determined according to modified consensus criteria: stage 0 (no TDP-43 pathology); stage 1 (TDP-43 pathology present only in amygdala); stage 2 (TDP-43 pathology extends into the entorhinal cortex or hippocampus, but not the neocortex); and stage 3 (TDP-43 pathology extends into the neocortex) [1,35]. For analyses, stages 0–1 were classified as LATE-NC negative (reference), and stages 2–3 as LATE-NC positive.

**Neocortical Lewy bodies:** Neocortical Lewy bodies were assessed using immunohistochemistry with antibodies specific to alpha-synuclein (Zymed LB 509; 1:50; pSyn, 1:20 000; Wako Chemicals) from the substantia nigra, amygdala, and entorhinal, middle temporal, midfrontal, inferior parietal and anterior cingulate cortices and classified as present if Lewy body was detected in any of the neocortical region including middle temporal, midfrontal, and inferior parietal cortices [5].

**Cerebrovascular pathologies and tissue injury:** Atherosclerosis severity (none-severe) was determined based on the number of plaques, the extent of involvement of each artery by plaques, and the degree of vascular occlusion on bisecting vessels at the Circle of Willis and their proximal branches [7]. Arteriolosclerosis severity (none-severe) was graded from the anterior basal ganglia [7]. Both vascular pathologies were dichotomized as none-mild versus moderate-severe in the analyses [7,36,37]. Chronic gross infarcts were identified during gross examination and confirmed their presence by microscopically [41]. Chronic microinfarcts were identified from a minimum of nine brain regions during microscopic evaluation and treated as present if microinfarcts were detected in any of assessed region [5].

**Supplementary Table 1:** Multivariable-adjusted associations of CAA but also other brain pathologies (ADNC, LATE-NC, LBs, and vascular) with the log10-transformed cortical brain iron, Rush Memory and Aging Project (N=626). Values in cells represent estimated coefficients (SE, *P value*).

| Predictor | Outcome: Cortical brain iron | |
| --- | --- | --- |
|  | Model A | Model B |
| CAA presence | 0.027 (0.011, 0.015) | 0.022 (0.010, 0.041) |
| ADNC (Intermediate/high) | 0.014 (0.010, 0.139) |  |
| Amyloid-β |  | -0.001 (0.006, 0.875) |
| Tau-tangles |  | 0.023 (0.005, <0.001) |
| LATE-NC (Stage 2/3) | 0.018 (0.009, 0.051) | 0.009 (0.009, 0.329) |
| LBs | 0.005 (0.013, 0.697) | -0.0001 (0.012, 0.993) |
| Arteriolosclerosis | -0.009 (0.009, 0.332) | -0.013 (0.009, 0.166) |
| Atherosclerosis | 0.004 (0.009, 0.656) | 0.006 (0.009, 0.527) |
| Cerebral infarcts | 0.003 (0.008, 0.679) | 0.004 (0.008, 0.574) |

Model A and B were adjusted for age at death, sex, and education.

**Supplementary Fig. 1:** Illustrative cases. Histological sections from representative participants. A. Case 1 is a female participant, (age-at-death is 85.2 years), with an intermediate ADNC and absence of CAA pathology in the inferior temporal cortex. B. Case 2 is a female participant (age-at-death is 86.6 years), with an intermediate ADNC and mild CAA pathology in the inferior temporal cortex. C. Case 3 is a female participant (age-at-death is 84.8 years), with an intermediate ADNC and moderate CAA pathology in the inferior temporal cortex. D. Case 4 is a female participant (age-at-death is 87.2 years), with an intermediate ADNC and severe CAA pathology in the inferior temporal cortex. Images represent Meguro staining on the sections of the inferior temporal cortex and reflect the elevation of iron with CAA severity. E. Case 4 also represents the iron deposition in the CAA vessel walls (arrow). Scale bars: 100µm (A-D) and 50 µm (E).

**
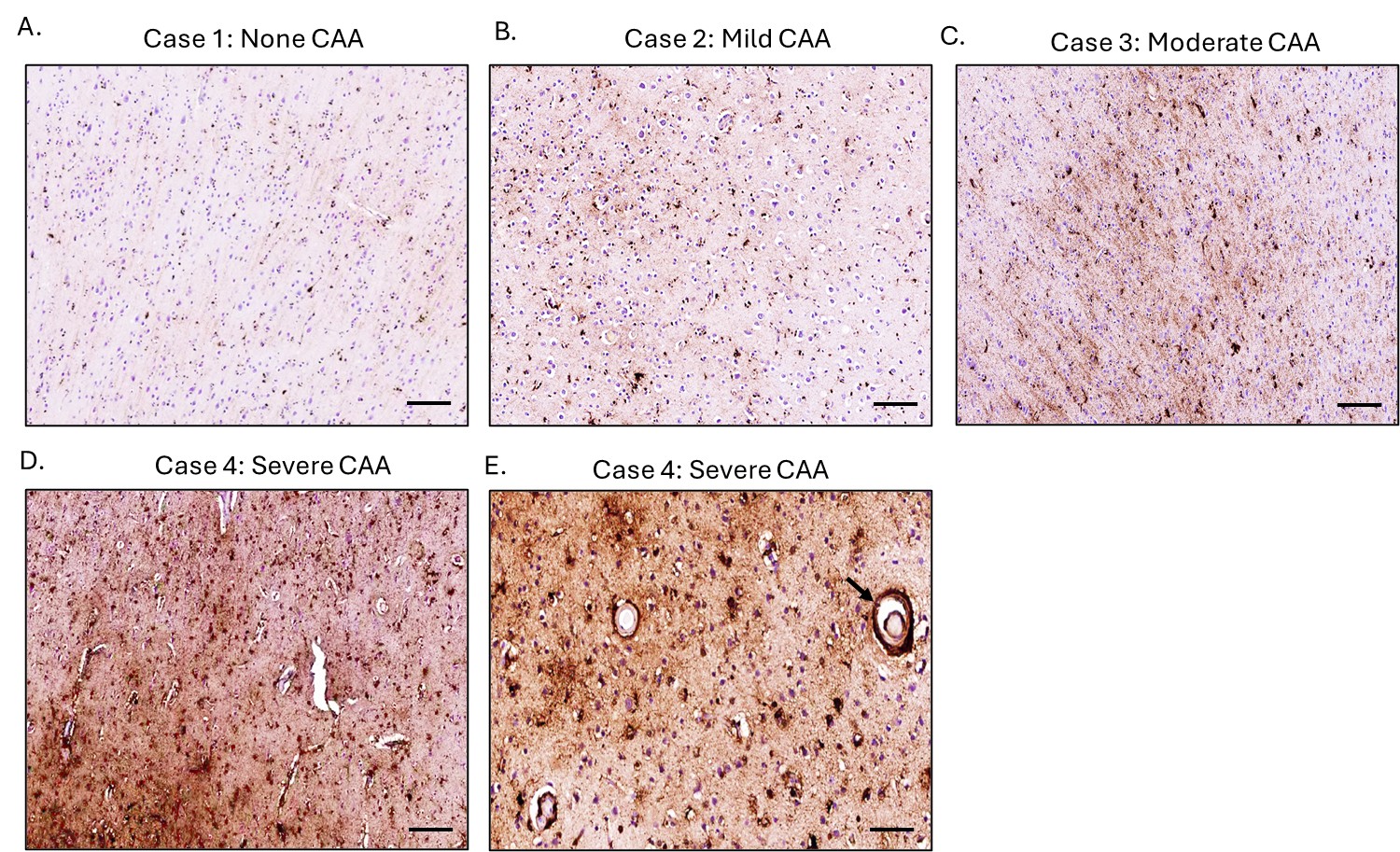
**

**Supplementary Fig. 2.** Mean estimated trajectories of change in global cognition and five cognitive domains in the years before death, by increasing CAA severity. Estimated trajectories are derived from six separate linear mixed models, with time before death approximated using natural cubic splines. Each model included: log_10_-transformed brain iron levels; CAA severity; covariates (demographics, mode of cognitive assessment, ADNC, LATE-NC, LBs, arteriolosclerosis, atherosclerosis, and cerebral infarcts); and the interactions of all variables with the splines time function. Curves represent the mean estimated trajectories (solid lines) and 95% confidence intervals (indicated with shadings) of an average study participant profile (a woman, aged 90 years at death, with 15 years of education, who had intermediate ADNC likelihood and chronic infarcts but no other pathologies).


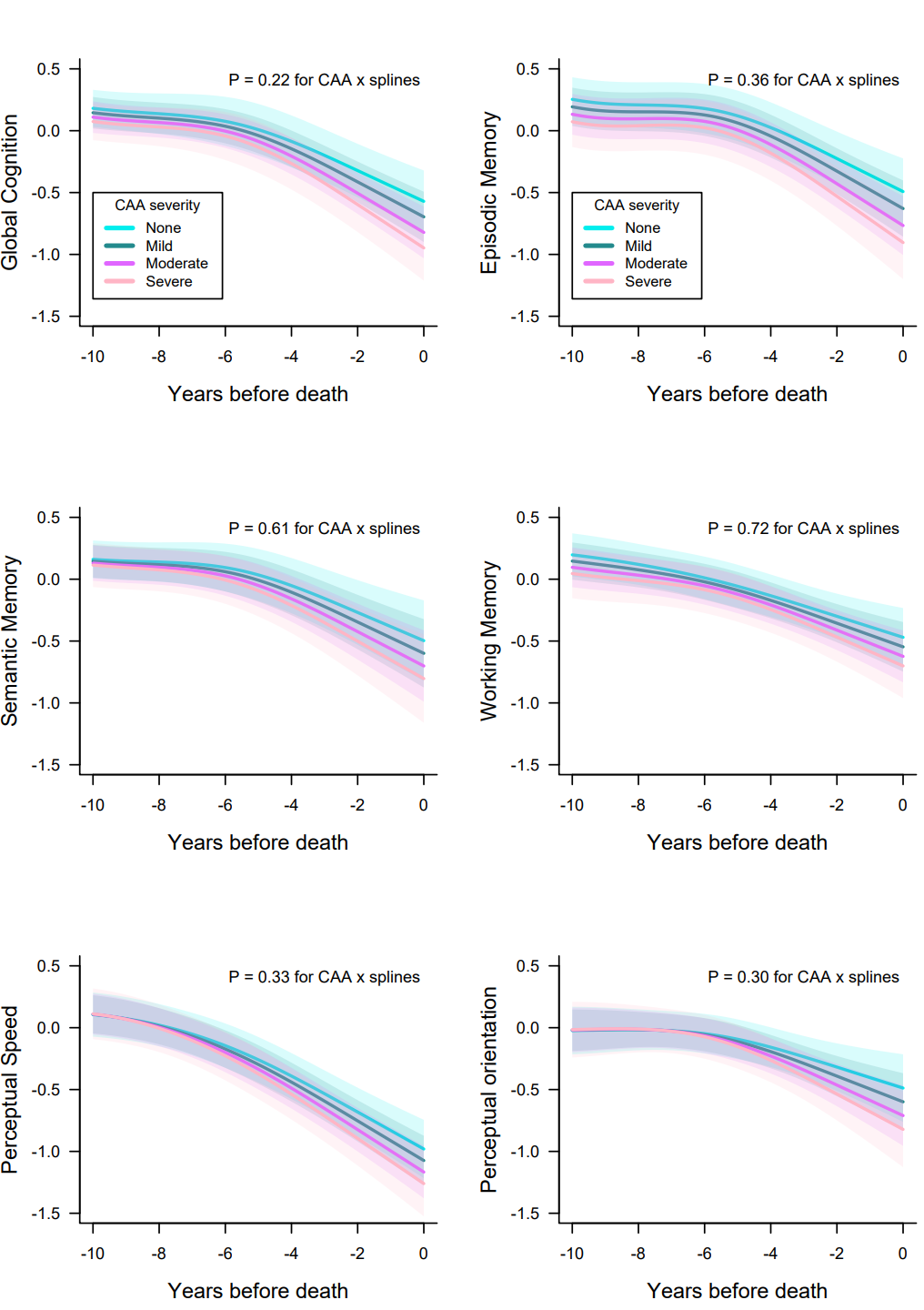


**Supplementary Fig. 3**. Mean estimated trajectories^a^ of change in global cognition in the years before death, by increasing levels of cortical brain iron and CAA severity, Rush Memory and Aging Project (N=626).

^a^Estimated trajectories are derived from six linear mixed models, with time before death approximated using natural cubic splines. Each model included: log_10_-transformed brain iron levels; CAA severity; Iron by CAA interaction; covariates (demographics, mode of cognitive assessment, ADNC, LATE-NC, LBs, arteriolosclerosis, atherosclerosis, and cerebral infarcts); and the interactions of all variables with the splines time function. Curves represent the mean estimated trajectories (solid lines) and 95% confidence intervals (indicated with shadings) of an average study participant profile (a woman, aged 90 years at death, with 15 years of education, who had ADNC and chronic infarcts but no other pathologies).


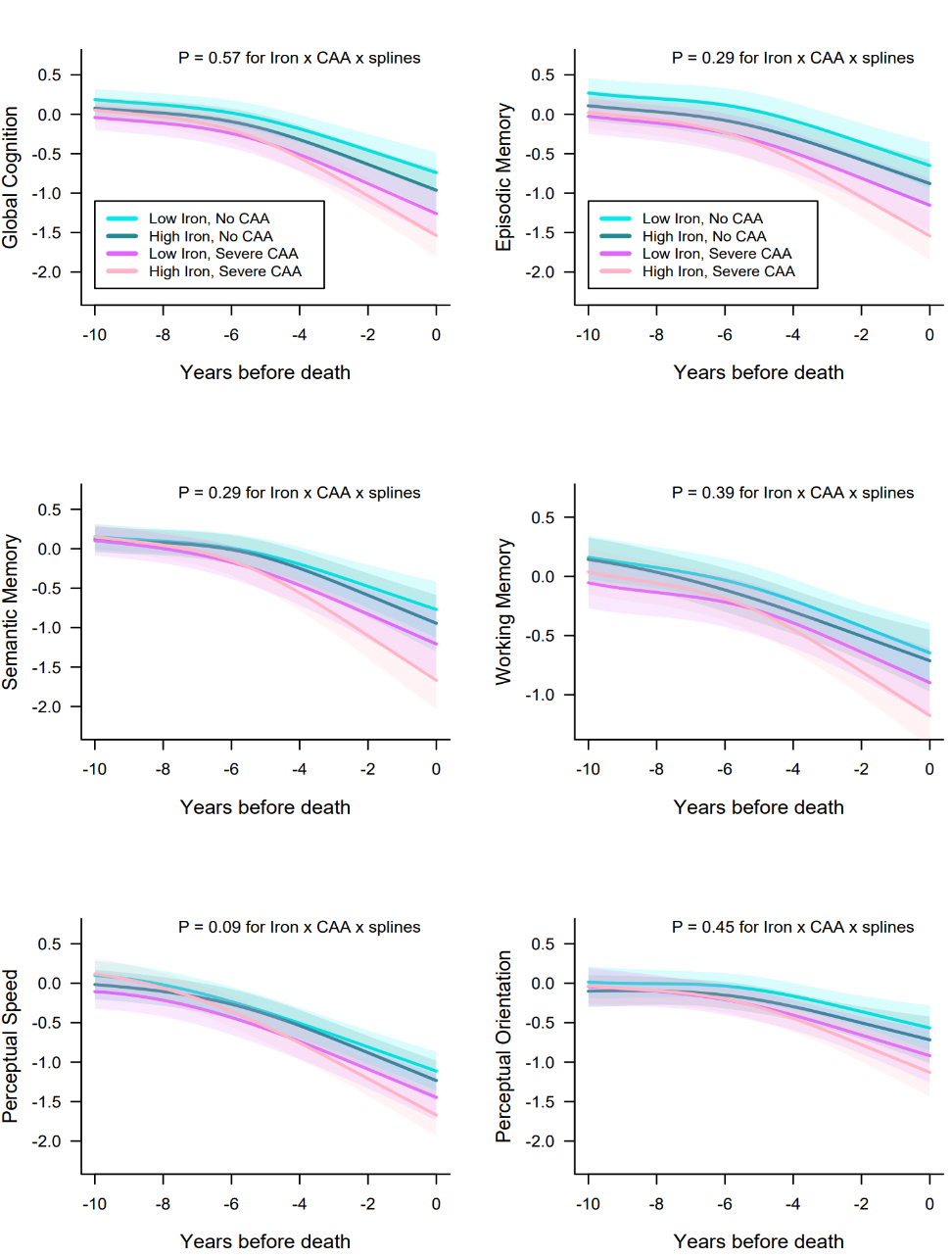

Supplement: Supplementary file 1 — Supplementary file1 (DOCX 1039 KB) [file 401_2026_3056_MOESM1_ESM.docx]
